# Transcatheter Pulmonary Valve Replacement by Hybrid Approach Using a Novel Polymeric Prosthetic Heart Valve: Proof of Concept in Sheep

**DOI:** 10.1371/journal.pone.0100065

**Published:** 2014-06-13

**Authors:** Ben Zhang, Xiang Chen, Tong-yi Xu, Zhi-gang Zhang, Xin Li, Lin Han, Zhi-yun Xu

**Affiliations:** 1 Department of Cardiothoracic Surgery, Changhai Hospital, Second Military Medical University, Shanghai, China; 2 Centre of Cardiovascular Surgery, Guangzhou General Hospital of Guangzhou Military Region, Guangzhou, China; 3 Department of Cardiology, Changhai Hospital, Second Military Medical University, Shanghai, China; Sapienza University of Rome, Italy

## Abstract

**Background:**

Since 2000, transcatheter pulmonary valve replacement has steadily advanced. However, the available prosthetic valves are restricted to bioprosthesis which have defects like poor durability. Polymeric heart valve is thought as a promising alternative to bioprosthesis. In this study, we introduced a novel polymeric transcatheter pulmonary valve and evaluated its feasibility and safety in sheep by a hybrid approach.

**Methods:**

We designed a novel polymeric trileaflet transcatheter pulmonary valve with a balloon-expandable stent, and the valve leaflets were made of 0.1-mm expanded polytetrafluoroethylene (ePTFE) coated with phosphorylcholine. We chose glutaraldehyde-treated bovine pericardium valves as control. Pulmonary valve stents were implanted in situ by a hybrid transapical approach in 10 healthy sheep (8 for polymeric valve and 2 for bovine pericardium valve), weighing an average of 22.5±2.0 kg. Angiography and cardiac catheter examination were performed after implantation to assess immediate valvular functionality. After 4-week follow-up, angiography, echocardiography, computed tomography, and cardiac catheter examination were used to assess early valvular function. One randomly selected sheep with polymeric valve was euthanized and the explanted valved stent was analyzed macroscopically and microscopically.

**Findings:**

Implantation was successful in 9 sheep. Angiography at implantation showed all 9 prosthetic valves demonstrated orthotopic position and normal functionality. All 9 sheep survived at 4-week follow-up. Four-week follow-up revealed no evidence of valve stent dislocation or deformation and normal valvular and cardiac functionality. The cardiac catheter examination showed the peak-peak transvalvular pressure gradient of the polymeric valves was 11.9±5.0 mmHg, while that of two bovine pericardium valves were 11 and 17 mmHg. Gross morphology demonstrated good opening and closure characteristics. No thrombus or calcification was seen macroscopically.

**Conclusions:**

This design of the novel ePTFE transcatheter pulmonary valve is safe and effective to deploy in sheep by hybrid approach, and the early valvular functionality is good.

## Introduction

Since the first sucessful clinical application of transcatheter pulmonary valve replacement (TPVR) was reported by Bonhoeffer and colleagues in 2000 [1], TPVR has steadily advanced and become readily adopted for the patients with stenotic or regurgitant pulmonary valve diseases, especially for patients with congenital heart disease (CHD) who had surgical pulmonary valvectomy or transannular pulmonary patches [2], [3].

However, the available prosthetic pulmonary valves are restricted to bioprosthesis which currently have various problems. First, most of the prospective patients for TPVR are young, the deterioration of bioprosthesis will lead to reoperation which means more agony for the young patients. Second, the sizes of currently available bioprosthetic valves are not suitable for use in all concerned patients due to anatomic variability [4]. Moreover, valved stent deployment during TPVR has been proved to be responsible for traumatic injury to bioprosthetic valve leaflets [5], [6], which concievably impact on the durability of bioprosthetic valves.

Polymeric prosthetic heart valve (PPHV) is a so-called “biomechanical valve” which is thought as a combination of the advantages of mechanical and bioprosthetic valves: long-term durability and no necessity for permanent anticoagulation [7], and as a promising alternative to bioprosthetic valves for TPVR [8]. Recently, Metzner et al [9] have demonstrated an excellent outcome of polyurethane valves with a self-expanding stent in the pulmonary position of sheep in a period of 4 weeks.

Currently, clinically used transcatheter pulmonary valve stents are disigned for percutaneous therapy. With currently available stents, it is not possible to treat all concerned patients. So far, patients with small or distorted peripheral vessels and patients with wide or severely calcified and kinked RVOTs are not suitable for percutaneous therapy. New stent designs and hybrid approaches are desired to overcome these problems.

In this study, we designed a novel trileaflet polymeric transcatheter pulmonary valve (PTPV) with a balloon-expandable stent, and the valve leaflets were made of 0.1-mm expanded polytetrafluoroethylene (ePTFE) coated with phosphorylcholine. The aim of this study was to evaluate the feasibility and safety of this novel valve for TPVR in sheep by a hybrid approach and assess the function of this valve during a 4-week period using angiographic, hemodynamic, echocardiographic, and macroscopic analyses and histologic assessment.

## Materials and Methods

### Ethics Statement

All the animal experimental protocols were approved by the ethics committee of Changhai Hospital, Shanghai. All animals received care in compliance with the Guide for the Care and Use of Laboratory Animals.

### Phosphorylcholine (PC) Coating of ePTFE Membranes

The 0.1-mm ultramicroporous ePTFE membranes (Gore-Tex, WL Gore and Associates, Inc., Flagstaff, AZ) were coated with a derivative of PC as previously described [10], [11]. Briefly, 2-methacryloxyethyl phosphorylcholine (MPC, 1 mol eq) and N-butyl methacrylate (BMA, 2 mol eq) were polymerized by a solution free radical process using azobisisobutyronitrile (0.2 mol eq) as initiator. Polymer (MPC–BMA) was dispersed in ethanol to give a 1% (w/v) mixture. The 0.1-mm ePTFE membranes were coated by a dip coating process, whereby the membranes were immersed in the solution and withdrawn at a rate of 30 cm/min. The coated membranes were dried at room temperature for 1 h and sterilized by autoclave.

### Valved Stent Construction

**Figure 1:**
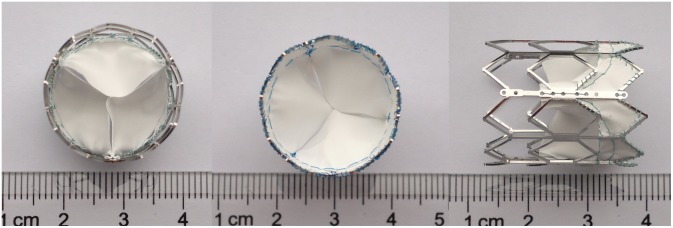
Views of the polymeric prosthetic pulmonary valve. Top view, bottom view, and side view of the balloon-expandable cobalt-chromium alloystent (length, 24.6 mm; diameter, 20 mm) containing a 3-leaflet ePTFE valve.

The balloon-expandable stent was made of cobalt-chromium alloy. Bare stents were 24.6 mm in length and 20 or 23 mm in diameter when fully expanded. The valve leaflets of PPHV were made of the PC-coated ePTFE membrane, which was trimmed into 3 identically shaped valve leaflets according to the valve model profile and sutured onto the stent using 7–0 polypropylene threads (Johnson & Johnson, New Brunswick City, NJ). The bottom one-third of the stent is covered with the PC-coated ePTFE sealing cuff designed to reduce the potential for paravalvular leaks (Fig. 1).

### Reference Valve

Bovine pericardium was used for the leaflets of reference valves. The bovine pericardium was processed by removal of surface fat tissue, agitated digestion with 0.01% trypsin for 8 hours, subsequent cross-linking with 0.6% glutaraldehyde for 36 hours, and, finally, repeated washing with 2%L-glutamine (to eliminate glutaraldehyde toxicity)[12]. The bovine pericardium was mounted on the stents by the similar method as PTPV.

### Animals

Ten healthy male sheep, 5 to 8 months old and weighing an average of 22.5±2.0 kg, were used. No cardiac murmurs were detected. Preoperative electrocardiogram, chest film, and echocardiogram revealed no abnormalities.

### Preoperative Preparation and Anesthetization

Animals were fasted for 12 hours and water deprived for 8 hours before surgery. Hair was removed from the right side of the chest wall. After intramuscular injection of ketamine (10–15 mg/kg, IM), diazepam (0.2 mg/kg, IM) and atropine (0.05 mg/kg, IM), a venous transfusion access on the foreleg was established and a 6-French leak-proof sheath was introduced percutaneously via the left femoral vein. Anesthesia was induced with fentanyl (2–4 ug/kg, IV), midazolam (0.05 mg/kg, IV), and vecuronium bromide (0.05 mg/kg, IV) and maintained with infusions of fentanyl (1–4 ug/kg·h) and propolfol (0.3 mg/kg·h). Ventilation was supported by use of a volume-limited ventilator with inspiratory volume of 10–15 ml/kg, respiratory rate of 18–24/min, and pressure support of 8–12cm H_2_O. Electrocardiogram, SpO2, and non-invasive blood pressure were routinely monitored throughout the whole procedure.

### Hybrid Approach and Implantation Procedure

**Figure 2:**
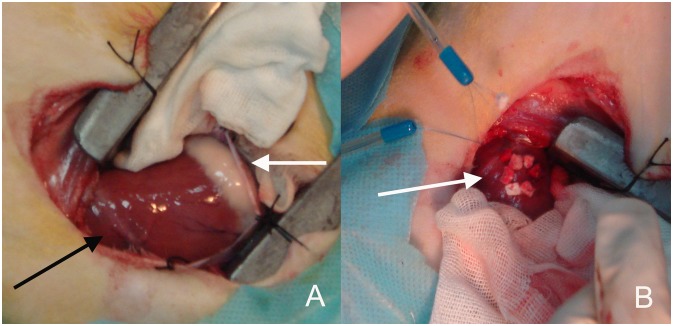
Views of the surgical approach. **A** The thoracic cavity was opened via the right anterolateral thoracotomy at the fourth intercostal space, and the right ventricular anterior wall and apex were exposed. Black arrow showing the left anterior descending coronary artery (LAD). White arrow showing the right atrioventricular groove. **B** Two felt strip-buttressed purse-string sutures were placed at the right ventricular apex almost 10 mm far away from LAD (white arrow).

The sheep were positioned in the left lateral decubitus. The thoracic cavity was opened via the right anterolateral thoracotomy at the fourth intercostal space. Then the right ventricular apex was exposed, and two felt strip-buttressed purse-string sutures using 5–0 polypropylene threads (Johnson & Johnson, New Brunswick City, NJ) were made at the right ventricular apex, almost 10 mm far away from the left anterior descending coronary artery (Fig. 2).

**Figure 3:**
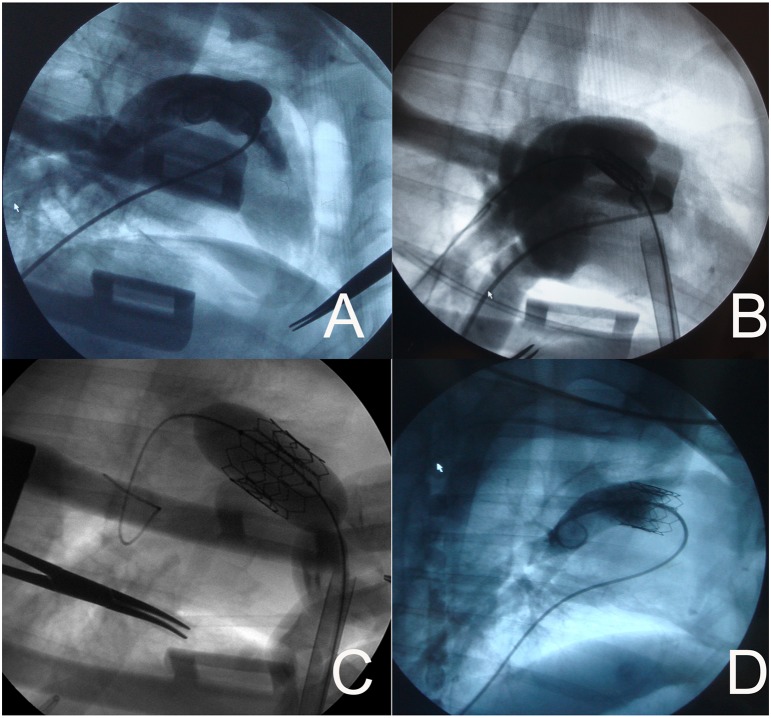
Valved stent implantation process (representative images). **A** Imaging of the pulmonary valve was performed to measure the pulmonary valve radius and location. **B** After the valved stent was advanced to the pulmonary valve via the 22F sheath, a right ventricular angiography was performed to confirm that the valved stent was at the optimal position. **C** The valved stent was fully balloon-expanded. **D** A pulmonary angiography showing correct position of the valved stent in the pulmonary position in an sheep model. No regurgitation was assessed.

**Figure 4:**
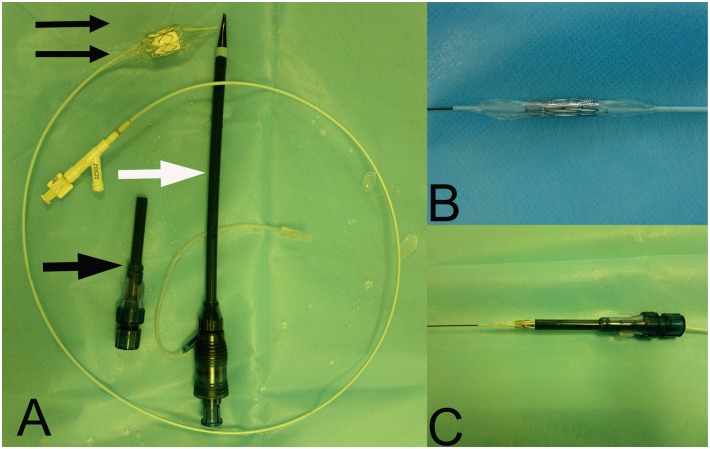
Views of the delivery system. **A**. Views of the 22F introducer sheath set (white arrow), the auxiliary short sheath (black arrow), and the 20 mm×3 cm high-pressure balloon catheter (double black arrows). **B** The valved stent was crimped symmetrically onto the inflatable portion of a balloon catheter. **C** Placement of valved stent into the auxiliary short sheath.

Heparin (1.5 mg/kg) was introduced via the sheath in the left femoral vein. A 6-French pigtail catheter (Cordis, Johnson and Johnson, New Brunswick City, NJ) was introduced through the femoral vein sheath, and imaging of the right ventricle and pulmonary valve were performed using digital subtraction angiography (DSA) (Siemens, Munich, Germany) (Fig. 3A). The pulmonary valve was identified, and its position was marked according to imaging results, and the radius of the pulmonary valve annulus was measured. A valved stent, size larger than the measured annulus, was selected to avoid paravalvular leaks and in consideration of the growth of the young sheep. Then the valved stent was crimped symmetrically using a specialized crimping device onto the inflatable portion of a 20 or 23 mm×3 cm high-pressure balloon catheter (volume driven) (Fig. 4). The balloon catheter loaded with valved stent was subsequently inserted into the auxiliary short sheath (Fig. 4). After that, the prepared right ventricular apex was punctured in the direction of the right ventricular outflow tract (RVOT), and an over-the-needle 7F introducer system (Arrows, Reading, Pa) was inserted. Under fluoroscopic guidance, a soft-tip 0.035-inch guide wire was first advanced into the right pulmonary artery and then transcatheter exchanged for an ultrastiff Back-up Meier wire (0.038 inches, 185 cm; Boston Scientific, Medi-tech, Watertown, Mass). After that, the 7F introducer was removed, and the 22F introducer sheath set (Fig. 4) was inserted over the guide wire during fluoroscopy. Then the 22F sheath and the guide wire were fixed, and the introducer was removed. The auxiliary short sheath and the prepared balloon catheter loaded with valved stent was inserted into the 22F sheath. Thereafter, the valved stent was advanced to the previously marked position of the pulmonary valve (Fig. 3B). After it was confirmed that the valved stent had been delivered to the optimal position, the valved stent was then balloon-expanded (Fig. 3C). The balloon was subsequently deflated, and the catheter and the 22F sheath were removed. Imaging of the artificial pulmonary valve and the right ventricle was performed by deploying a pigtail catheter via the left femoral vein (pressure 800 psi, total amount of contrast 20 ml, speed 20 ml/s) (Fig. 3D). A chest tube was placed and the chest was closed.

### Postoperative Treatment

All sheep received postoperative intramuscular injections of cefradine (25 mg/kg, 1/12 h) for 3 days and subcutaneous injections of low molecular heparin (60–80 IU/kg, 1/12 h) for 7 days. Oral aspirin (3 mg/kg·d) was given for the entire study period. One week after surgery, the sheep returned to the animal facility, where their general health was checked daily.

### Angiography

Angiography was performed before the procedure to locate the precise position of the native pulmonary valve, after the procedure, and at 4-week follow-up to confirm the appropriate position and verify the function of the implanted valve. (Fig. 3).

### Echocardiographic and Hemodynamic Measurements

Transthoracic echocardiography (TTE) was performed at 4-week follow-up. Right ventricular pressure, pulmonary artery pressure, and peak-peak transvalvular pressure gradient (PTPG) were collected before and after the implantation and at 4-week follow-up by cardiac catheterization.

### Computed Tomography

The 64-row computed tomography was performed at 4-week follow-up to confirm the appropriate position and intact configuration of the stent.

### Laboratory Blood Chemistry Measurements

Erythrocyte and white blood cell counts, hematocrit, free hemoglobin, platelet count, coagulation parameters, and levels of aspartate aminotransferase, alanine aminotransferase, lactate dehydrogenase, bilirubin, creatinine, serum calcium and phosphorus were checked 4 weeks after implantation and compared with preoperative values.

### Valved Stent Retrieval

One randomly selected sheep with polymeric valve was euthanized and the valved stent was explanted 4 weeks after implantation. Before harvesting, heparin (100 IU/kg IV) was administered. Autopsy with macroscopic and histologic examination of heart, lungs, liver, kidneys, and spleen was performed. The valved stent was rinsed with a saline solution to remove excess blood, and then was examined macroscopically, histologically, and by electron microscopic analysis. The histology stains included standard hematoxylin and eosin (H&E) to detect tissue deposition, and von Kossa staining to detect calcium deposition on the leaflets.

### Electron Microscopic Analysis

Samples for electron microscopy analysis were taken from the explanted valve leaflets and were rinsed in a solution of paraformaldehyde. For scanning electron microscopy (SEM), the tissue samples were dehydrated in a graded acetone series, and critical-point dried. Specimens were then glued to metal stubs, coated with gold, and examined in a Hitachi S-520 scanning electron microscope (Hitachi, Ltd., Chiyoda, Tokyo). For transmission electron microscope (TEM), the tissue samples were postfixed in osmium tetroxide and embedded in araldite. Ultrathin sections were stained with uranylacetate and lead citrate and observed with a Hitachi H-7650 transmission electron microscope (Hitachi, Ltd., Chiyoda, Tokyo).

### Statistics

Values are presented as means±standard deviations. Changes in right ventricular pressure, pulmonary artery pressure, and transvalvular gradients (before, right after, and 4 weeks after implantation) were compared using repeated measurements or Friedman test. Statistical analyses were performed by using SPSS 16.0 software (SPSS, Inc, Chicago, Ill). P values of less than 0.05 were considered statistically significant.

## Results

Implantation was successful in 9 sheep. Angiography at implantation showed one 20 mm PTPV failed to be located at orthotopic position and the stent was in RVOT. All the other prosthetic valves demonstrated orthotopic position and exhibited normal open and close functionality and no stenosis or insufficiency. Six sheep were implanted with 20 mm PTPV, and two sheep with 23 mm PTPV. The other 2 sheep were implanted with reference valve (20 mm and 23 mm valve, respectively).

The mean diameter of the pulmonary annulus was 16.8±1.4 mm and 17.5±1.1 mm as revealed by TTE and angiography, respectively. Mean surgery duration was 82±10.6 minutes. The mean x-ray exposure time was 7.5±1.6 minutes. The mean stent diameter (except the unsuccessful one) measured by angiography after the procedure was 20.5±1.6 mm (range: 18–23 mm).

In one sheep, postoperative chest drainage was above normal. The bloody chest drainage was 80 ml 3 h after surgery, and 160 ml 24 h after surgery. The chest tube was extracted 48 h after surgery. One sheep developed pneumothorax postoperatively, which was self-healing 48 h after surgery. Vomit and aspiration happened to one sheep before extubation during the surgery. The sheep developed cough and pneumonia postoperatively, and was successfully cured by antibiotic, expectorant, and chest physiotherapy for one week. The other sheep were uneventful postoperatively. All 10 sheep including the unsuccessful one survived and did well in the 4-week follow-up. The 4-week follow-up data excluded the unsuccessful one.

**Figure 5:**
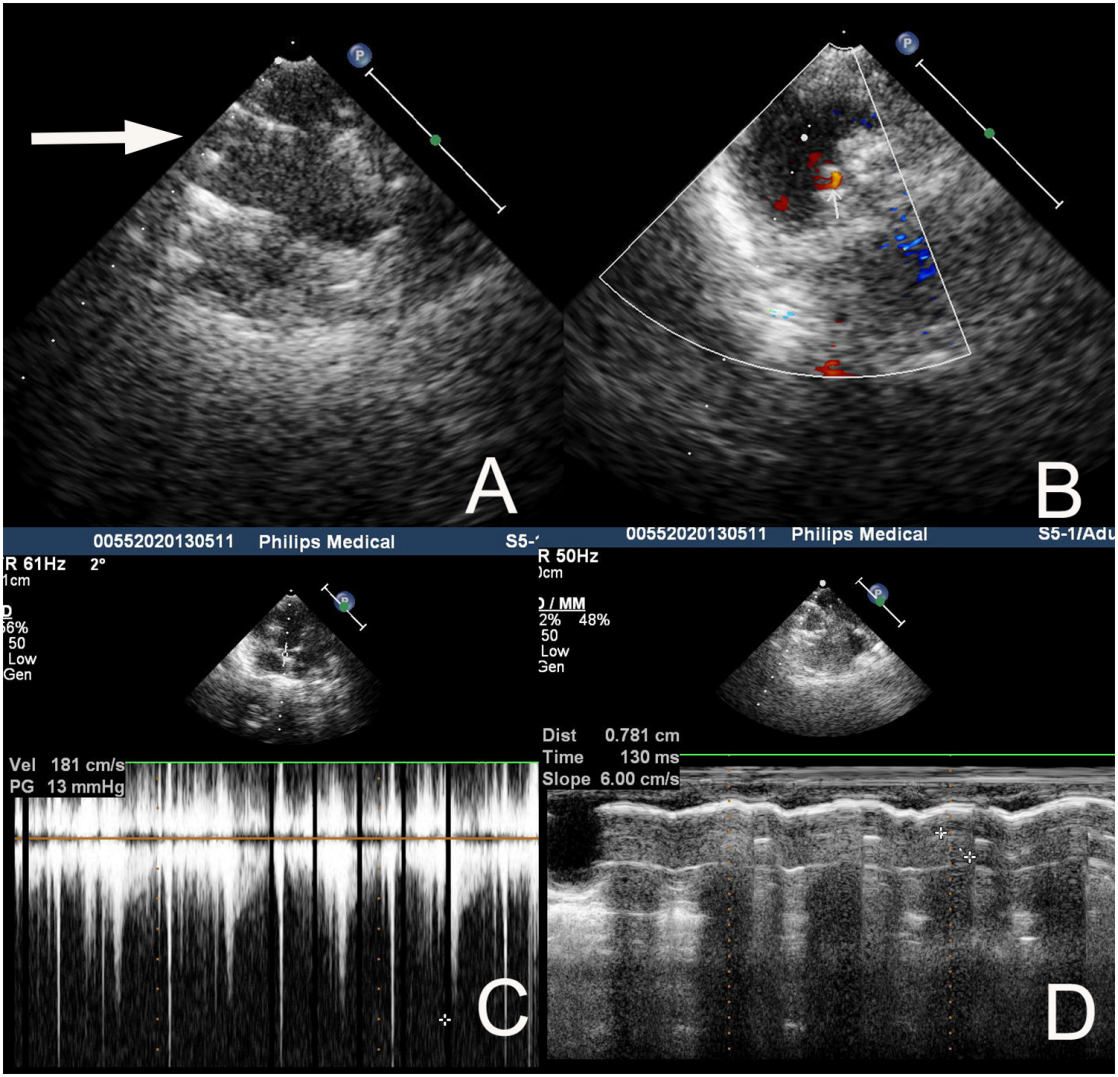
Echocardiography 4 weeks after implantation from a representative. sheep with a 20 mm PTPV. **A** The two parallel lines of echo enhancement showed appropriate position and open shape of the stent (white arrow). **B** Color Doppler ultrasonography revealed no regurgitation or paravalvular leakage. **C** Doppler ultrasonography revealed the peak-peak transvalvular pressure gradient of the stented valve was 13 mmHg. **D** The motion distance of the valve cusp was 0.781 cm measured by Doppler ultrasonography, meaning normal function of the valve leaflet.

Echocardiography 4 weeks after implantation (Fig. 5) showed all the prosthetic valves exhibited normal functionality and no significant insufficiency, paravalvular leakage, or valve excrescence. Left ventricular and right ventricular function and dimensions appeared normal. PTPG of the polymeric valves was 14.1±6.6 mmHg, while that of two reference valves were 9 and 16 mmHg.

Four-week angiography demonstrated good opening and closure characteristics in all 9 sheep after 4 weeks. Paravalvular leakages were not observed. The mean stent diameter measured by 4-week angiography was 20.1±1.5 mm (range: 18.5–22 mm).

**Figure 6:**
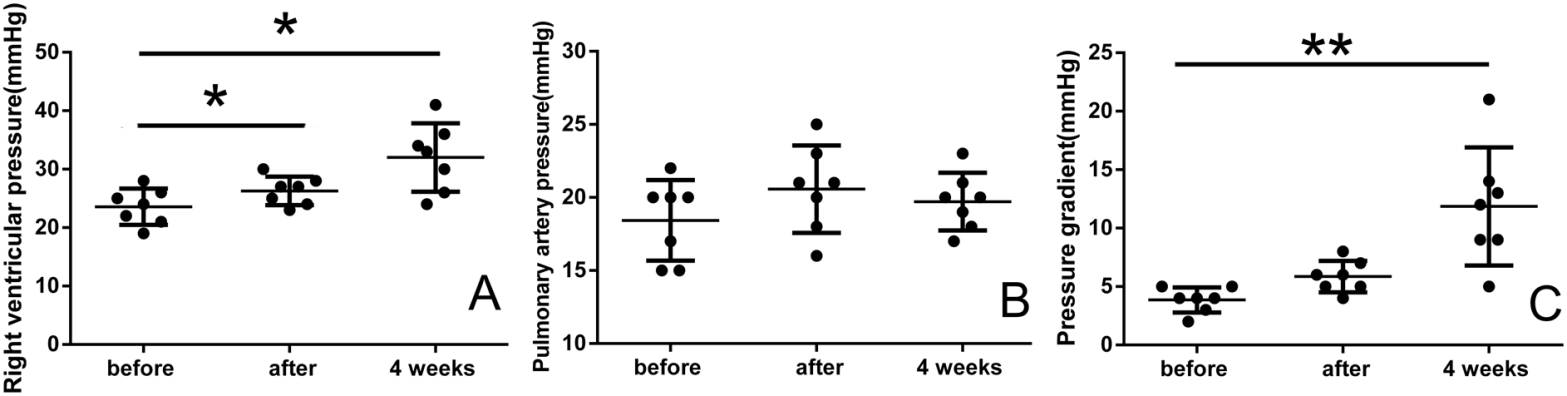
Hemodynamic data before, immediately after, and 4 weeks after implantation. Right ventricular systolic pressure (A), pulmonary artery systolic pressure (B), and peak-peak transvalvular pressure gradient (C) were assessed. vs. before group, *P<0.05, **P<0.01.

The results of cardiac catheterization revealed that PTPG of the polymeric valves was 3.9±1.1 mmHg before implantation, 5.9±1.3 mmHg right after implantation and 11.9±5.0 mmHg at follow-up (P<0.01). While, PTPG of the two reference valves were 2 and 4 mmHg before implantation, 4 and 7 mmHg right after implantation, and 11 and 17 mmHg at follow-up. Right ventricular pressure, pulmonary artery pressure, and PTPG of the polymeric valves before and after the implantation and at 4-week follow-up are shown in Figure 6.

**Figure 7:**
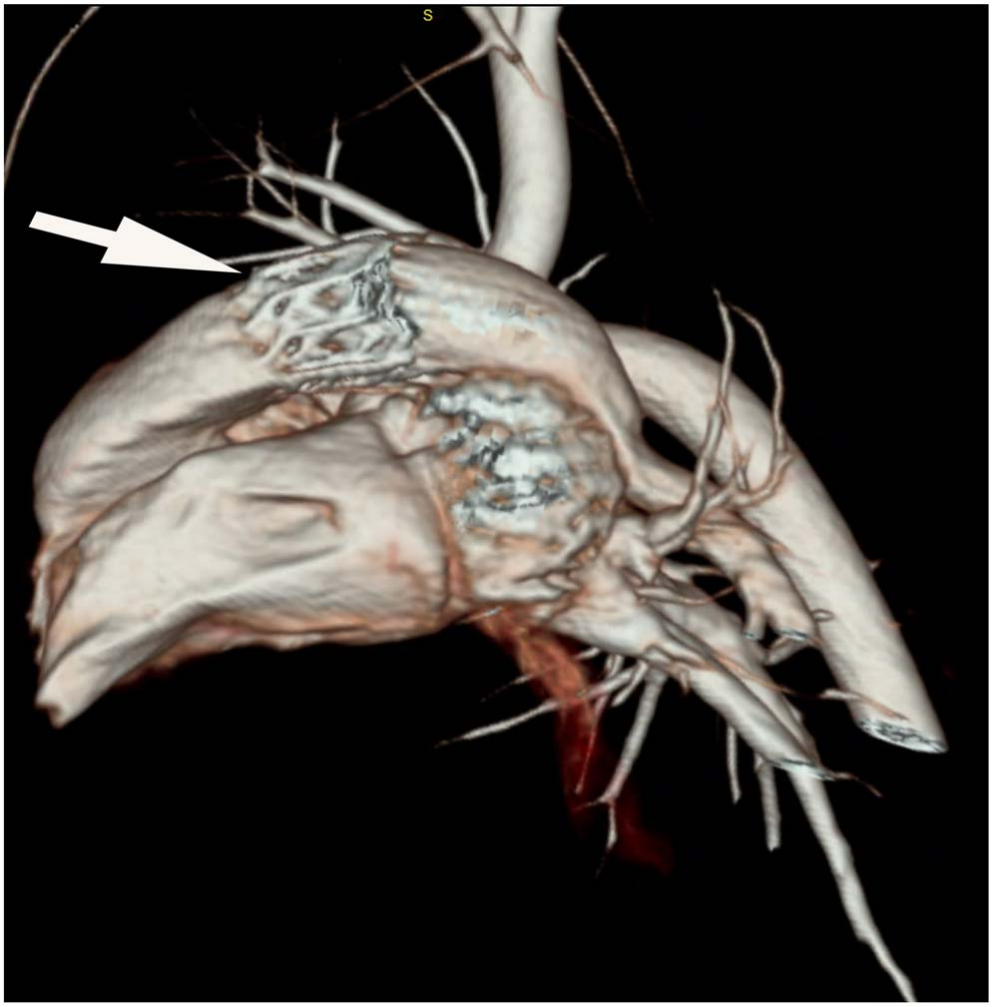
The 3D reconstruction CT image 4 weeks after implantation from a representative sheep with a 20 mm PTPV. The image demonstrated orthotopic position and no deformation of the stent (white arrow).

CT 4 weeks after implantation demonstrated orthotopic position and no evidence of stent fracture or deformation of all 9 stents (Fig. 7).

Blood parameters of all sheep did not show any significant changes from preoperative values.

**Figure 8:**
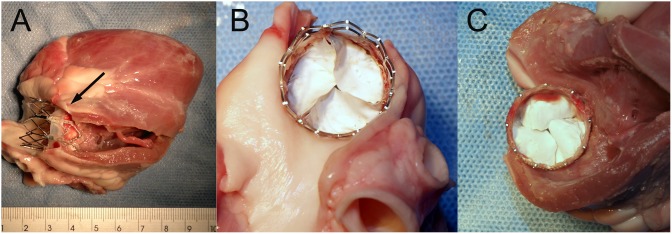
Gross morphology of the ePTFE valved stent explanted from a sheep 4 weeks after surgery. **A** The native pulmonary valve can be seen (arrow), confirming the correct position of the valved stent. **B** The outflow side of ePTFE valved stent showed the leaflets were thin without significant tissue deposits. **C** The inflow side of ePTFE valved stent showed slight fibrous overgrowth at the bottom of the leaflets, in the commissural areas and on the sealing cuff.

Postmortem examination of the randomly selected sheep confirmed the correct position of the valved stent and that the native pulmonary valve was stuck between the pulmonary wall and the stent. No paravalvular defects were visible. The valve leaflets were thin, pliable, and competent, without thrombus or calcification. No significant tissue deposits were noted in the outflow side of the ePTFE valved stent, while the inflow side of the ePTFE valved stent showed slight fibrous overgrowth at the bottom of the leaflet, in the commissural areas and on the sealing cuff. Cardiac structures were unscathed (Fig. 8).

**Figure 9:**
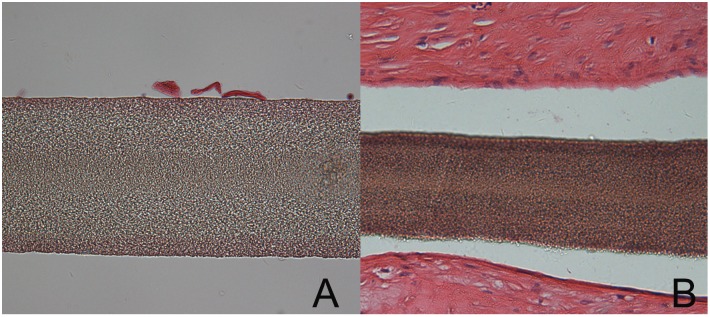
Histologic images of the ePTFE valve leaflets explanted from a sheep 4 weeks after surgery. **A** The surface of ePTFE leaflet was smooth, without obvious cell or tissue infiltration. The ePTFE membrane showed multilayered structure.(HE×400). **B** The surface of ePTFE on the sealing cuff revealed fibrous overgrowth, which attached loosely to the ePTFE membrane which itself had no obvious cell or tissue infiltration.(HE×200).

On microscopic evaluation (Fig. 9), there was no cellular infiltration or structural deterioration of the ePTFE membrane noted. Slight fibrous overgrowth was observed on the sealing cuff and at the bottom of the leaflets. The fibrous overgrowth attached loosely to the ePTFE membrane which itself had no obvious cell or tissue infiltration. No evidence of calcification of the stented valve including the valve leaflets and sealing cuff was found after von Kossa staining. The surrounding tissue of the pulmonary artery showed normal H&E staining and von Kossa staining.

**Figure 10:**
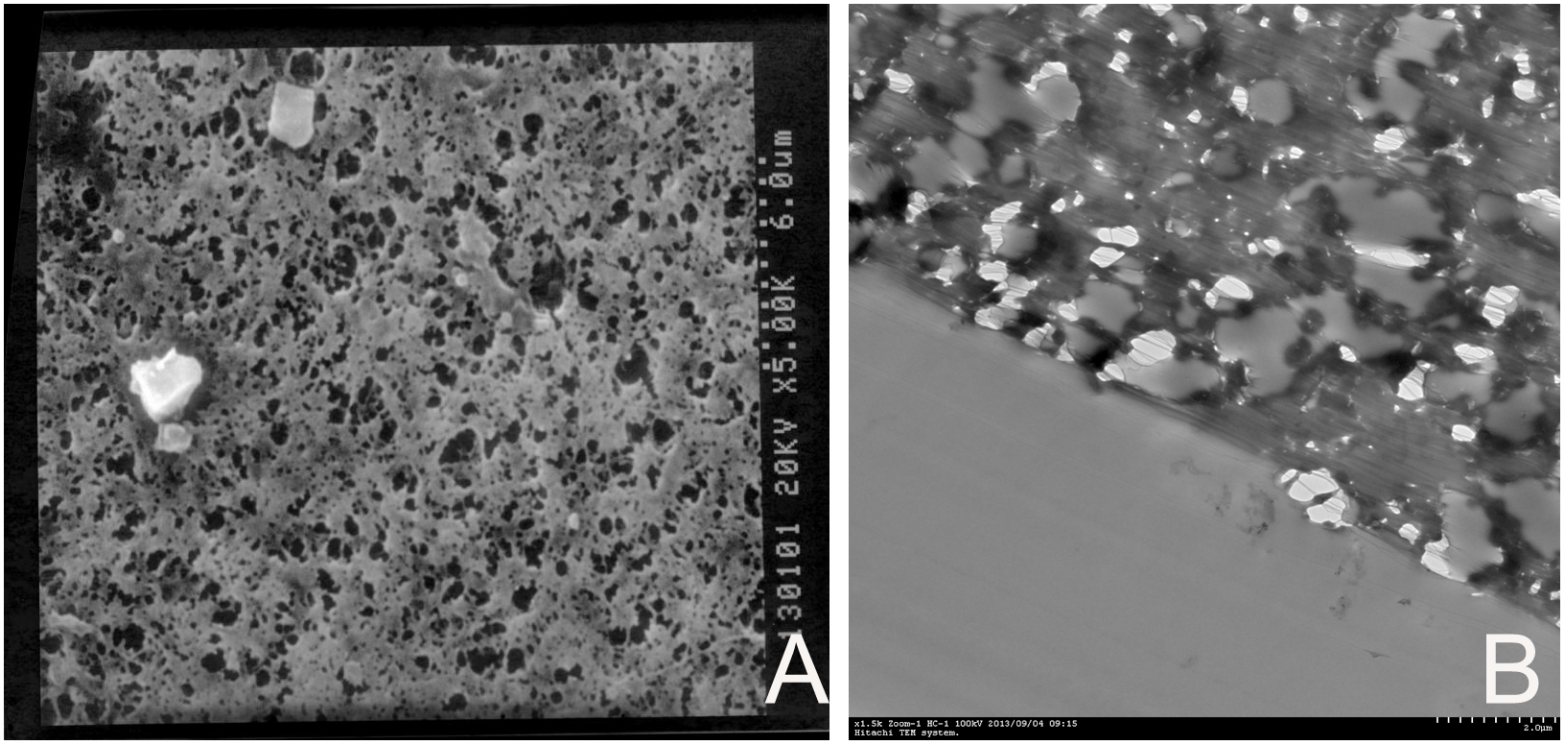
Electron microscopic images of the ePTFE leaflets explanted from a sheep 4 weeks after surgery. **A** Scanning electron microscopic appearance of the outflow side of a ePTFE valve cusp showing polyporous structure of ePTFE and no obvious cell or tissue attachment (×5000). **B** Transmission electron microscopic appearance of the outflow side of a ePTFE valve cusp showing polyporous structure of ePTFE and no obvious cell or tissue infiltration. (×1500).

No cell or tissue attachment/infiltration was found in the SEM or TEM images (Fig. 10). Pathological findings showed no abnormality in the organs of the sheep.

## Discussion

Since 2000, TPVR technology has steadily advanced [2], [13], [14]. Clinically, TPVR appears to offer significant benefits over standard surgical procedures for patients following surgery for CHD, including tetralogy of Fallot, pulmonary atresia and other surgical procedures requiring reconstruction of RVOT [2]. Not only is the process superior in smaller incisions, less postoperative pain, shorter hospital stay, and lower costs, but the short and medium follow-up data show satisfactory clinical effectiveness and good freedom from reoperation, and demonstrate that TPVR can potentially reduce the lifetime number of open heart operations [2], [14], [15]. Bonhoeffer et al [2] successfully performed 155 procedures with no early deaths, only 4 procedural complications, and a good survival rate of 96.9% after 83 month.

However, clincally available stented valves for TPVR are restricted to transcatheter bioprosthetic valves (TBVs), which have some significant defects. Firstly, the target population for TPVR are almost the young. The unsatisfactory durability of bioprosthesis certainly will increase the pain of the young patients. Secondly, the leaflets of TBVs, especially with balloon expandable valved stents, are proved to be injured after crimped and uncrimped during implantation. Zegdi et al [5] report the microscopic analysis of 4 Sapien-Edwards TBVs, which have been crimped and deployed in humans or ex vivo. Collagen fibers fragmentation and disruption in the valve leaflets could be found in all of them. The thickness of the leaflets was involved and areas of plasmatic insudation was found. These findings were also proved by animal experiment [6], in which the TBVs leaflets were implanted subcutaneously in rats for 12 weeks after crimped and uncrimped, and microscopic analysis revealed intense fragmentations of the collagen and elastic fibers and moderate invasion of macrophages, giant cells and signs of phagocytosis. These changes will conceivably impact on the long-term durability of TBVs. Thirdly, the resulting anatomic spectrum from pulmonary regurgitation after surgical intervention for CHD is broad. With currently available TBVs, it is not possible to treat all concerned patients. Moreover, with younger patients, especially children, the overall size of the currently available TBVs are too large. So, the indications for TPVR includes the conduit RVOT diameter should be between 16 mm and 22 mm and the weight should be more than 20 kg [16].

The advancement in the study of PPHV has brought emerging hope to these patients by remedying those weaknesses of TBVs. Over the past 20 years, significant advances in material science has resulted in new materials with improved properties, which have the advantages of good durability and antithrombotic performance and being easily engineered and designed to reproduce the geometry and haemodynamic characteristics of natural valves, making them potential materials of choice in the development of valve substitutes [17]. Moreover, the application of low-profile PPHV could be an advantage for TPVR because it is easy for PPHV to be folded and introduced into delivery catheters [17].

Recently, several transcatheter PPHVs [18], [19] have been designed and the results of in vitro test were good. Moreover, Metzner et al [9] have demonstrated an excellent valve function of polyurethane valves with a self-expanding stent in the pulmonary position of 7 sheep in a period of 4 weeks. Gross morphology demonstrated good opening and closure characteristics and the valve leaflets were thin and pliable without indurations or calcification.

ePTFE is a hydrophobic and polyporous polymer, with chemically inert, low friction, and low tissue affinity. The main advantages of ePTFE is its electronegative surface charge, which mimics the normal endothelium and would supposedly decrease its thrombogenicity [20]. This low tendency to **produce thrombotic events**, together with suitable mechanical properties and fatigue resistance, made this material a good choice for PPHV. In 1990s, Nistal et al [20] designed a PPHV in which the cuspal material was made of ePTFE with low porosity and the material covering the valve frame was made of ePTFE with high porosity, and sucessfully implanted ten PPHVs in the tricuspid position in sheep for 3 to 42 weeks with no postoperative anticoagulation. They found that the PPHVs had a moderate calcification rate and that calcium deposits appeared to be always related to the presence of ePTFE with high porosity. No evidence of valve thrombosis was found in 9 animals. Microscopy disclosed a complete lack of infiltrating cells within the cuspal material made of ePTFE with low porosity, however, those parts made of ePTFE with high porosity did show infiltration by host cells and calcium. These results presented that PPHV made of ePTFE had excellent anti-thrombotic function and the substitution of the ePTFE with high porosity might eventually eliminate these aforementioned problems.

In this century, a novel ultramicroporous ePTFE (UEPTFE) became a popular material for PPHV, especially in the pulmonary valve position. This extremely low porosity (distance of fiber nodes <1 um) of UEPTFE makes cellular or fibrinous adherence more difficult and is theoretically resistant to pannus formation or calcification. The monocusp valve [21], bicuspid valve [22], and trileaflet valve [23] made of UEPTFE have successfully been implanted in men with CHD for pulmonary reconstruction, and have demonstrated satisfactory clinical effects. Ando et al [23] reported their ten-year experience with handmade trileaflet UEPTFE valved conduit used for pulmonary reconstruction in 139 patients with no postoperative anticoagulation. Estimated freedom from conduit explantation was 88.0%, and pulmonary insufficiency was less than or equal to mild in 75.0% at 10 years. All valves maintained their motion, and no evidence of valve thrombus was noted.

These achivement of UEPTFE valve in the pulmonary reconstruction has brought hope for further developing a transcatheter UEPTFE pulmonary valve.

However, some problems with UEPTFE valves remain to be resolved. Concerns have been raised about leaflet thickening and decreased mobility caused by neointimal and fibrous overgrowth after valve repair with UEPTFE membrane [24], [25]. Given this, in this study, we used the PC-coating technique to provide a more biocompatible and nonthrombogenic surface for UEPTFE membrane. PC is a zwitterionic head group with no overall charge found in cell membrane lipid bilayers, which is nonthrombogenic. A coating of PC derivatives onto polymetric materials has shown decreased platelet adhesion and neointimal hyperplasia to the polymer surface in many studies [10], [11], [26], [27]. The advantage of such an approach is that the blood that comes into contact with PC-coated surfaces maintains its homeostatic potential. After 4-week follow-up, we found the PPHVs with PC-coated UEPTFE showed good antithrombotic property and resistance to tissue affinity. However, mild fibrosis developed on the sealing cuff of this UEPTFE valve, and it spread to the commissural areas and the bottom of the leaflets. The stagnant flow in these areas might facilitate the apposition of fibrin and blood cell elements, and lead to fibrosis. The mild fibrosis didn’t influenced the valve function in period of 4-week follow-up, while further results remained to be observed.

There are two available surgical approaches for TPVR: transvascular or transventricular approach. Currently, clinically used surgical approach for TPVR is confined to transvascular approach. For patients with small or distorted peripheral vessels and patients with wide or severely calcified and kinked RVOTs, hybrid operation and transventricular approach will be the only option. So, developing a safe and minimally invasive hybrid approach is meaningful. Moreover, although less trauma, transvascular approach can bring two problems in sheep model: first, the diameter of peripheral vein in sheep is often small, which restricts the choice of valved stent size; second, the position and direction of heart and main artery are different between tetrapod sheep and bipedal human. The angles from inferior vena cava to right ventricle and from right ventricle to pulmonary artery in sheep are more sharp than those in human. So, it is difficult to deliver the sheath from femoral vein to pulmonary valve in sheep model [28], and it is easy to induce arrhythmia during this procedure [9]. In Metzner’s study [9], rhythm disturbances occurred in all animals. Likewise, in our preliminary test, we had tried transfemoral approach in sheep with a 14F sheath, but we failed to deliver the sheath to pulmonary valve because of the sharp turning from tricuspid valve to pulmonary valve in sheep, and the repeated procedure led to ventricular fibrillation and death. After autopsy, we found endocardium scathed in the anterior wall of right ventricle. So, in this study, we chose the hybrid method and transapical approach for all sheep. We found TPVR by the hybrid method and transapical approach with this novel ePTFE stented valve was safe and effective. No serious arrhythmia occurred, and cardiac structures were intact.

To avoid stent migration, a right choice of valved stent size and a good coaxality between valved stent and pulmonary artery root when expanding balloon are important. We suggest a ratio of 1.2∼1.3∶1 between the diameter of the valved stent and the pulmonary valve ring, and a projection position of RAO 20/CRA 20 (sheep in left lateral decubitus), in which the angle from right ventricular long axis to main pulmonary artery would be as straight as possible. In one sheep, the valved stent was located below the right position. We thought a bad coaxality was the reason.

In conclusion, TPVR with the novel PC-coated UEPTFE stented valves by the hybrid method and right ventricular apical approach in an ovine model is feasible and safe. The early valvular functionality of the novel UEPTFE stented valves are as good as bovine pericardium valves. The PC-coated UEPTFE valves have demonstrated good antithrombotic performance and low cell or tissue affinity. The medium-term results remain to be observed.
